# Molecular origins of synaptotagmin 1 activities on vesicle docking and fusion pore opening

**DOI:** 10.1038/srep09267

**Published:** 2015-03-20

**Authors:** Ying Lai, Xiaochu Lou, Jiajie Diao, Yeon-Kyun Shin

**Affiliations:** 1 Department of Biochemistry, Biophysics & Molecular Biology, Iowa State University, Ames, Iowa 50011, USA; 2 Center for Mitochondrial Biology and Medicine, The Key Laboratory of Biomedical Information Engineering of Ministry of Education, School of Life Science and Technology and Frontier Institute of Life Science, Frontier Institute of Science and Technology (FIST), Xi'an Jiaotong University, Xi'an 710049, P. R. China; 3 Biomedical Research Institute, Korea Institute of Science and Technology (KIST), Hwarangno 14-gil 6, Seongbuk-gu, Seoul 136-791, South Korea

## Abstract

Synaptotagmin 1 (Syt1), a major Ca^2+^ sensor in neuroexocytosis, utilizes SNARE- and membrane-binding to regulate vesicle fusion, a required process for neurotransmitter release at the synapse. However, the mechanism by which Syt1 orchestrates SNARE- and membrane- binding to control individual vesicle fusion steps is still unclear. In this study, we used a number of single vesicle assays that can differentiate intermediates of neuroexocytosis, to focus on Syt1 mutants that might impair Syt1-SNARE/PIP_2_ interaction, Ca^2+^-binding, or membrane penetration. Our results show that, although putative Syt1-SNARE/PIP_2_ coupling through the polybasic region of the C2B domain is critical for vesicle docking, its disruption does not affect content release. In contrast, Ca^2+^-binding and membrane-penetration mutants significantly reduce content release. Our results thus delineate multiple functions of Syt1 along the pathway of Ca^2+^-triggered exocytosis in unprecedented detail.

In the pre-synapse, Ca^2+^-triggered neurotransmitter release from synaptic vesicles is the key process for maintaining signal transduction in the neuronal system^1^. SNARE proteins, comprised of presynaptic t-SNAREs syntaxin 1A and SNAP-25 and vesicle v-SNARE VAMP2 (vesicle-associated membrane protein 2), function as the core fusion machinery in vesicle exocytosis^2^,^3^,^4^,^5^. For tight control of neurotransmitter release by Ca^2+^, another vesicle protein, synaptotagmin 1 (Syt1), has been identified as a major Ca^2+^ sensor^6^,^7^. It is believed that Syt1 plays a key role in synchronizing neurotransmitter release by sensing Ca^2+^ influx and subsequently triggering rapid fusion of the vesicle with the plasma membrane^1^,^7^,^8^,^9^,^10^.

At the molecular level, Syt1 contains an N-terminal transmembrane domain, a flexible linker region, and tandem Ca^2+^-binding C2 (C2A and C2B) domains^11^. Cytoplasmic C2A and C2B domains share a β-sandwich structure containing a bottom loop region that can bind two and three Ca^2+^ ions, respectively^12^,^13^,^14^. Syt1 can interact with t-SNARE and the ternary SNARE complex via the polybasic region on the C2B domain in both Ca^2+^-independent and -dependent manners^15^,^16^,^17^,^18^,^19^. Also, Syt1 could bind to the negatively charged lipid PIP_2_ (Phosphatidylinositol 4,5-bisphosphate) on the plasma membrane probably via the same polybasic region on the C2B domain in the absence of Ca^2+^, while the loop regions could penetrate into the acidic membrane upon Ca^2+^ binding^20^,^21^,^22^,^23^,^24^.

In functional studies, both SNARE and membrane binding by the C2 domains have proven essential in achieving vesicle fusion^18^,^25^,^26^,^27^,^28^,^29^,^30^. Mutations in the polybasic region resulted in the impairment of SNARE-mediated lipid mixing *in vitro* as well as Ca^2+^-triggered neurotransmitter release *in vivo*^21^,^31^,^32^. Mutations in the Ca^2+^ binding sites revealed that Syt1 is the Ca^2+^ sensor and that Ca^2+^ binding to the loop region is required for vesicle exocytosis^6^,^29^,^33^,^34^,^35^. Finally, the loss and the gain of function mutations on the loop region showed that its membrane penetration is a critical step for Ca^2+^-triggered membrane fusion^26^,^27^,^33^,^36^,^37^. It has recently been shown that membrane attachment through its transmembrane domain is important for Syt1 to function properly as a Ca^2+^-sensor^29^,^38^,^39^,^40^.

It is thought that vesicle fusion proceeds in at least three sequential steps: vesicle docking, hemifusion (or lipid mixing), and fusion-pore opening (or content release)^9^,^41^,^42^, and studies have shown that Syt1 might be involved in all three steps. Apparently, Syt1's interactions with the SNARE complex and with the membrane are responsible for these regulations. For example, the putative Syt1-t-SNARE/PIP_2_ interaction assists docking of vesicles to the plasma membrane^18^,^30^,^43^. Syt1's penetration into the membrane is likely to play roles in both lipid mixing and fusion-pore opening^6^,^26^,^27^,^29^,^33^,^34^,^35^,^36^,^37^. The Syt1-ternary SNARE interaction might regulate those later steps as well in response to the Ca^2+^ signal, although this has not been demonstrated experimentally. Because of experimental difficulties for previous ensemble fusion assays in resolving individual steps along the fusion pathway, many ambiguities still remain with respect to understanding the molecular origins of the Syt1's involvement in individual steps of Ca^2+^-triggered neuroexocytosis.

To address these issues, we generated polybasic region mutants, which are likely to impair the Syt1-SNARE/PIP_2_ interaction, Ca^2+^ binding site mutants to hamper its Ca^2+^ affinity, and loop region mutants to alter its membrane penetration capacity. With well-established single vesicle assays^40^,^44^,^45^,^46^ that, can resolve the vesicle fusion process into docking and content release reflecting fusion pore opening, we dissected the effect of these mutations on individual fusion steps. The results show that Ca^2+^-independent t-SNARE and PIP_2_ binding, mediated by the polybasic region of Syt1, is essential for vesicle docking. To our surprise, however, content release is regulated only by the Ca^2+^-dependent insertion of the loop region and Ca^2+^ binding sites of Syt1 into the membrane, and not at all by its SNARE/PIP_2_ interaction.

## Results

### Syt1 mutants and their SNARE interactions

**Figure 1:**
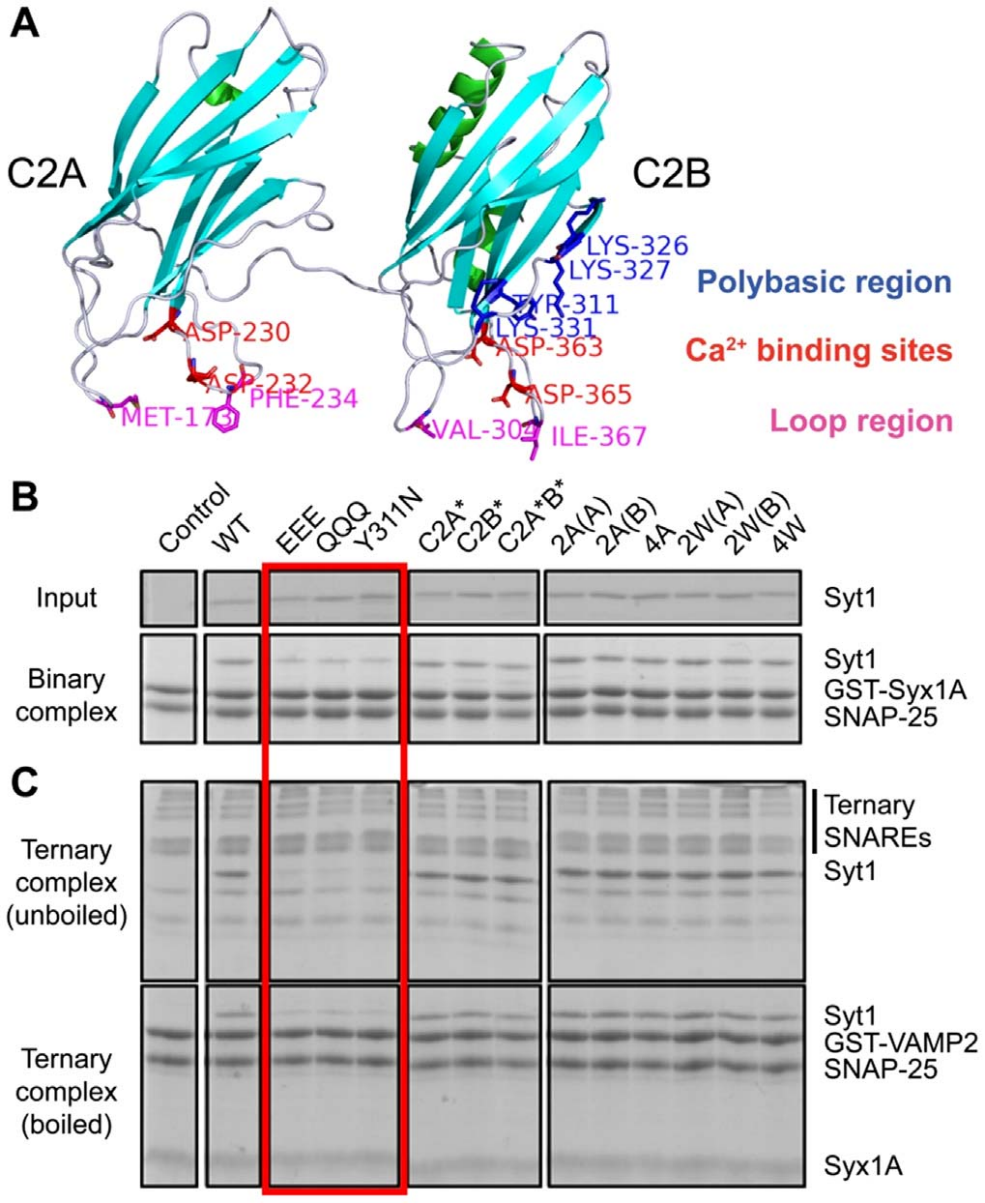
SNARE binding of Syt1 mutants. (A) Ribbon diagram of the cytoplasmic domain of Syt1 (C2AB). The model is derived from the crystal structural of the cytoplasmic domain of Syt1 (C2AB, PDB id: 2R83)^69^. The C2B domain was rotated about 180° in the horizontal plane relative to C2A and the linker between C2A and C2B to show the Ca^2+^ binding loops of both domains in the same plane. Two important Ca^2+^ binding residues (D230/D232) on the C2A domain and the other two Ca^2+^ binding residues (D363/D365) on the C2B domain are shown as red sticks. Four residues proposed to be important for membrane insertion (M173/F234 on C2A, V304/I367 on C2B) are shown in magenta. Four residues on the C2B domain (K326/K327/K331/Y311) that are proposed to bind the SNARE complex, are shown in blue. (B and C) GST pull-down assays of SNARE-Syt1 binding. Mutations in the polybasic region of the C2B domain (EEE, QQQ, and Y311N) hamper SNARE binding while the Ca^2+^ binding (C2A*, C2B*, and C2A*B*) and loop region mutations (2A(A), 2A(B), 4A, 2W(A), 2W(B), and 4W) mutations do not change the SNAREs binding capability of Syt1. The binary complex is formed between GST-tagged soluble syntaxin 1A (191–266) and His-tagged SNAP-25 and the ternary complex is formed among GST-tagged soluble VAMP2 (1–96), syntaxin 1A (191–266), and His-tagged SNAP-25.

Syt1 mutants were designed on the basis of three reported functions of Syt1 in synaptic vesicle exocytosis: its interaction with SNARE and/or PIP_2_, Ca^2+^ binding, and membrane penetration. To alter these features of Syt1, we generated three groups of previously well-characterized Syt1 mutants. First, we intended to disrupt the SNARE/PIP_2_-binding polybasic region of the C2B domain by changing three basic amino acids Lys326, Lys327, and Lys331 to either acidic amino acid Glu (EEE), or neutral amino acid Gln (QQQ)^21^,^31^,^32^. We note however that there might be other binding modes between SNAREs and Syt1 (see Refs. 19,47). Another mutant, Y311N, was generated, because, although Y311 is a residue buried inside the polybasic region, this mutation has been shown to affect Syt1's binding to binary t-SNARE^18^,^48^, thereby hampering vesicle docking (blue in Fig. 1A). We also note though that these mutations might disrupt the Syt1 structure, thereby impacting functions. Such disruption is especially concerning for partially buried position 311(Ref. 48). The circular dichroism (CD) spectra however show that the overall folding was not affected significantly by the Tyr to Asn mutation at this position (Supplementary Fig. S1). Second, to block the Ca^2+^ binding capability, we mutated the amino acids Asp230 and Asp232 in the C2A domain to Ala (C2A*), the amino acids Asp363 and Asp365 in the C2B domain to Ala (C2B*), or generated all four mutations in the C2A and C2B domains (C2A*B*)^6^,^14^,^29^,^33^,^34^,^35^,^49^ (red in Fig. 1A). Third, we generated loop region mutants to reduce the membrane penetration ability of Syt1. We mutated amino acids Met173 and Phe234 in C2A to Ala (2A(A)), amino acids Val304, Ile367 in C2B to Ala (2A(B)), or generated all four mutations in C2AB (4A)^27^,^33^,^50^. Conversely, to enhance membrane penetration ability, we changed these amino acids to Trp instead of Ala (2W(A), 2W(B), 4W)^26^,^27^,^33^,^36^,^37^ (magenta in Fig. 1A).

Because it has been shown that Syt1 binding to the binary complex of t-SNAREs syntaxin 1A and SNAP-25 plays a role in vesicle docking prior to the rise of the Ca^2+^ level^18^, we first examined the Syt1 binding to the t-SNARE complex. The polybasic region mutants EEE, QQQ, and Y311N showed significant reduction of binding to the binary t-SNARE complex in the absence of Ca^2+^ (Fig. 1B). To further understand molecular mechanism of the t-SNARE-Syt1 interaction, we performed the GST pull-down assay in the present of 200 μM inositol 1,4,5-trisphosphate (IP_3_) to mimic PIP_2_ in solution. Our results showed that IP_3_ reduces the interaction between wild-type Syt1 and the binary t-SNARE complex somewhat (Supplementary Figs. S2B and S2C), indicating that Syt1 binding to PIP_2_ might compete with t-SNARE binding somewhat. Controls BSA and Ca^2+^ did not affect the interaction between Syt1 and the binary t-SNARE complex (Supplementary Figs. S2B and S2C), indicating that Syt1-binary t-SNARE interaction is specific and Ca^2+^-independent.

On the other hand, it has been postulated that Syt1 binding to the ternary SNARE complex regulates Ca^2+^-triggered opening of fusion-pores^7^. The polybasic region mutations reduced Syt1 binding to the ternary SNARE complex in the presence of Ca^2+^ (Fig. 1C). As controls, adding BSA or removing Ca^2+^ did not affect the interaction between Syt1 and the ternary SNARE complex (Supplementary Figs. S2D and S2E). IP_3_ (200 μM) did not alter the interaction between wild-type Syt1 and the ternary SNARE complex in the presence of 500 μM Ca^2+^ (Supplementary Figs. S2D and S2E). With these mutants we are now ready to test, with the single vesicle fusion assay whether or not there is direct correlation between Syt1-t-SNARE coupling and vesicle docking and, more importantly, between Syt1-ternary SNARE complex coupling and content release.

In contrast to these polybasic region mutations, neither the mutations in the Ca^2+^-binding sites nor those in the membrane-penetrating loops affected the Syt1's ability to bind the binary t-SNARE complex in the absence of Ca^2+^ or the ternary SNARE complex in the presence of Ca^2+^ (Figs. 1B and 1C).

### Polybasic region mutations in Syt1 reduce PIP_2_-binding and vesicle docking

To study the effect of the Syt1 mutants on individual fusion steps, we reconstituted wild-type Syt1 or its mutants and VAMP2 (molar ratio of 1:1) onto a population of vesicles (v-vesicles) (Supplementary Fig. S3). Premixed t-SNAREs (syntaxin 1A: SNAP-25 = 1:1.5) were reconstituted into another population of vesicles (t-vesicles) (Supplementary Fig. S3A). The wild-type Syt1 and its mutants had all similar reconstitution efficiencies when reconstituted together with VAMP2 to v-vesicles (Supplementary Fig. S3B).

**Figure 2:**
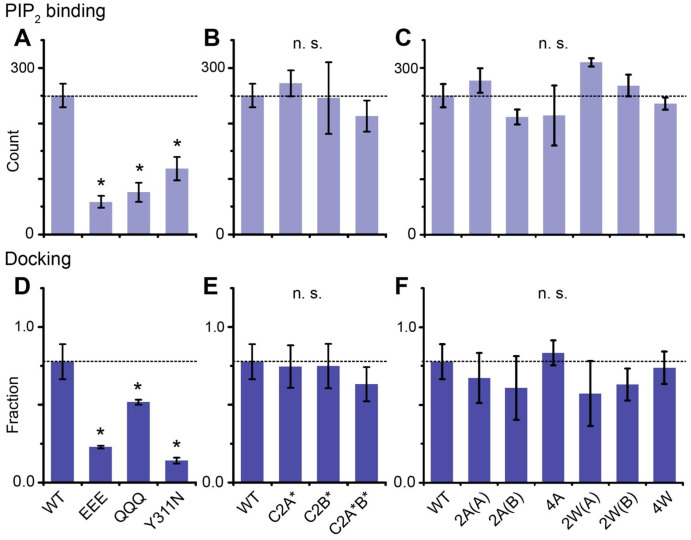
Interaction between the polybasic region of the C2B domain and PIP_2_ is important for vesicle docking. (A–C) PIP_2_ binding abilities of wild-type Syt1 and its mutants with alterations at the polybasic region, Ca^2+^ binding sites, and the loop region. (D–F) Single vesicle docking probabilities of wild-type Syt1 and its mutants with alterations at the polybasic region, Ca^2+^ binding sites, and the loop region. The fraction is defined as the number of docked t-vesicles divided by the number of immobilized v-vesicles. Results are shown as the mean ± S.D. (*P < 0.05, n = 3, and n.s. means ‘not significant').

Previously, we had shown that both binary t-SNARE and PIP_2_ play roles in Syt1-mediated vesicle docking^30^. Therefore, we performed the single vesicle membrane-binding assay to examine Syt1's binding to SNARE free vesicles (Supplementary Fig. S4A). In this assay, SNARE-free vesicles with 2 mol% PIP_2_ were immobilized (or tethered) on the surface by the avidin-biotin conjugation (see Materials and Methods section), Syt1- or its mutants-reconstituted vesicles doped with 2 mol% fluorescence acceptor lipid DiD (Syt1-vesicles) were loaded, and the number of Syt1-vesicles docked (or bound) to the SNARE-free vesicles in the imaging area (45 × 90 μm^2^) were counted. We found that only the polybasic region mutants showed impaired binding to the PIP_2_-containing vesicles by as much as 60% in the absence of Ca^2+^ (Fig. 2A and Supplementary Fig. S5), showing that Syt1 binding to the negatively charged PIP_2_ might contribute to vesicle docking, confirming our previous results^30^.

Then, we tested the effect of these mutations on vesicle docking by applying the single vesicle docking assay with t-vesicles containing DiD and v-vesicles containing fluorescence donor lipid DiI (2 mol% each) (Supplementary Figs. S4B and S4C)^40^,^51^,^52^. Our results showed that polybasic region-disrupted mutants Syt1 EEE and Y311N exhibited reduced vesicle docking by as much as ~70% when compared with wild-type Syt1. The QQQ mutant, however, had a mild inhibition by ~30% (Fig. 2D). Unlike the polybasic region mutants, the Ca^2+^ binding site and loop region mutants had negligible effects on vesicle docking (Figs. 2E and F). Similar to our previous work using solution single-vesicle assay, when PIP_2_ was removed from t-vesicles, vesicle docking is reduced significantly (Supplementary Fig. S6)^30^, suggesting that the Syt1-PIP_2_ interaction may well be the dominant force for vesicle docking. When the control IP_3_ or BSA was injected into the chamber together with t-vesicles, vesicle docking was not affected (Supplementary Fig. S6). Although our results may not be sufficient to pinpoint the detailed binding mechanism, they establish a direct correlation between Syt1-SNARE/PIP_2_ coupling and vesicle docking, and show that the polybasic region of Syt1 plays a role in vesicle docking.

### Ca^2+^ binding and the penetration of the loop region of Syt1 into the membrane are essential for content release

Ca^2+^ triggered neurotransmitter release at the synapse requires a fusion-pore encompassing the vesicle membrane and the plasma membrane. To study the effect of various Syt1 mutations on the fusion-pore opening step, we first examined the Ca^2+^ triggered insertion of Syt1 into the membrane. This time PIP_2_ was removed from SNARE-free vesicles and Syt1-vesicles were injected into the chamber in the presence of 500 μM Ca^2+^. We found that both Ca^2+^ binding site mutants and alanine mutants on the loop region showed impaired vesicle docking (Supplementary Fig. S7), consistent with the previous report that Ca^2+^ bridges Syt1 and the membrane and both C2A and C2B domains of Syt1 bind to the membrane in the presence of Ca^2+^ (Ref. 43).

**Figure 3:**
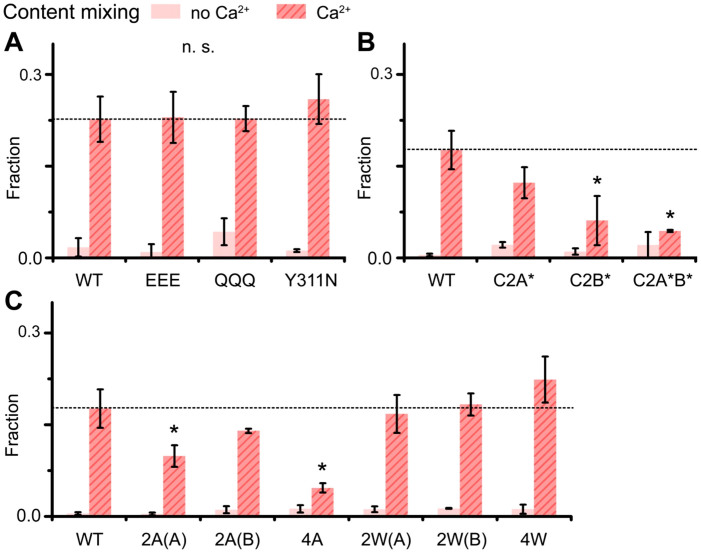
Content mixing of wild-type Syt1 and its mutants. The bar graphs represent single vesicle content mixing events within 1 min. of the fusion reaction. Pink bars represent the cases without Ca^2+^ while red bars represent the cases with 500 μM Ca^2+^. Content mixing probabilities are for wild-type Syt1 and its mutants in the polybasic region (A), for the Ca^2+^ binding sites (B), and for the loop region (C). The fraction is defined as the number of content mixing events divided by the number of docked vesicles. Results shown represent the mean ± S.D. (*P < 0.05, n = 3 and n.s. means ‘not significant').

Then we applied the single vesicle content mixing assay and monitored the diffusion of sulforhodamine B from a v-vesicle to an empty t-vesicle, that results in a sudden increase of the fluorescence signal due to dilution-induced fluorescence dequenching^40^,^46^ (Supplementary Fig. S8A). We used v-vesicles reconstituted with Syt1 and VAMP2 in a 1:1 molar ratio for wild-type Syt1 and its mutants, although the vesicles containing Syt1 and VAMP2 at the 1:4 molar ratio also exhibited robust content mixing (Supplementary Fig. S9). The polybasic region mutants EEE, QQQ, and Y311N showed no difference in term of the fusion percentage among docked vesicles from the wild-type Syt1 in Ca^2+^-triggered content release (Fig. 3A and Supplementary Fig. S8B). We note that the disruption of polybasic region by these mutations could still affect neurotransmitter release in fast (submillisecond) time scale after the Ca^2+^ influx *in vivo*. However, the Ca^2+^ binding sites mutants Syt1-C2A*, Syt1-C2B*, and Syt1-C2A*B* exhibited a decrease in Ca^2+^-triggered content release by 30, 60, and 70%, respectively (Fig. 3B and Supplementary Fig. S8C). The results indicate that Ca^2+^ binding to the C2 domain is important for Ca^2+^-triggered content release. We notice that the mutation in C2B is more severely disruptive than those in C2A, consistent with previous findings^25^.

Next, we tested loop region mutants that either reduce or enhance the membrane penetration ability of the loops, depending on the side-chain size of the corresponding amino acids. We observed an anticipated inhibition of content release for alanine mutants (2A(A), 2A(B), 4A) by 40%, 20%, 70% (Fig. 3C and Supplementary Fig. S8D), respectively. The results thus indicate that membrane penetration of Syt1 is important for opening fusion pores. In contrast, we observed no enhancement for two tryptophan mutants (2W(A), 2W(B)), although the 4W mutant was able to increase content mixing by 20% (Fig. 3C and Supplementary Fig. S8D). As controls, in the absence of Ca^2+^ none of these mutants supported content mixing effectively (pink bars in Fig. 3 and dotted lines in Supplementary Fig. S8).

Our results thus show that the polybasic region that mediates Syt1-SNARE/PIP_2_ coupling plays a role in vesicle docking but not in the final fusion step. It appears, however, that Ca^2+^-binding and the loop penetration into the membrane are important elements in content release although they have minimal effect on vesicle docking.

## Discussion

**Figure 4:**
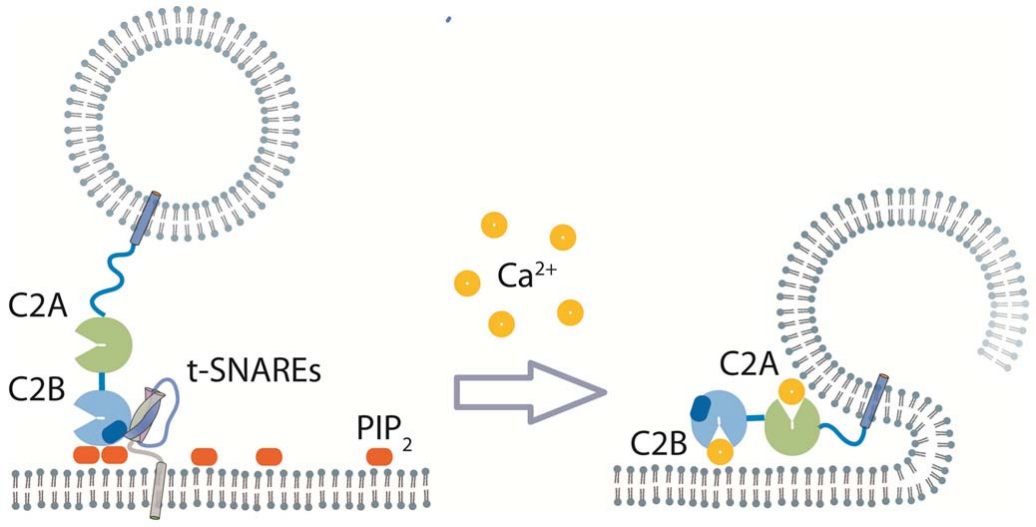
A mechanistic model for multiple synaptotagmin 1 functions along the vesicle fusion pathway. Before Ca^2+^, the polybasic region of the C2B domain interacts with t-SNAREs and PIP_2_ molecules to promote docking of synaptic vesicles. Upon calcium arrival, the membrane association and insertion of Ca^2+^-bound C2A and C2B domains become critical for content release.

In this work, our single vesicle fusion assay revealed that mutations in the polybasic region in Syt1 cause reduced SNARE and PIP_2_ binding and result in an apparent decrease in vesicle docking, but produce little change in the content release. It has been long speculated that Syt1-SNARE interaction plays a critical role in Ca^2+^-triggered exocytosis^25^. Our results show that the coupling between the polybasic region of Syt1 and SNARE/PIP_2_ is limited to vesicle docking (Fig. 4) and does not extend its influence on to the final content release step.

However, the Syt1-ternary SNARE complex coupling might still play an important role in regulating Ca^2+^-triggered exocytosis. A current mechanistic model predicts that complexins, a family of small presynaptic proteins, bind to the SNARE complex and inhibit full zippering, thereby clamping membrane fusion^53^,^54^,^55^,^56^,^57^,^58^. It has been thought that Syt1 would unclamp the complexin clamp in the presence of Ca^2+^, requiring Syt1's binding to the SNARE complex^53^,^54^. It thus appears that Syt1-ternary SNARE coupling is required for replacing the complexin clamp. In a separate note, since polybasic region mutants cannot completely block the Syt1/ternary SNARE interaction (Fig. 1 and Supplementary Fig. S2) we cannot rule out the possibility that there might be another unidentified region mediating the Syt1/ternary SNARE interaction besides the polybasic region^19^,^47^.

Although the Syt1-SNARE/PIP_2_ interaction has little influence on fusion pores, our results show that Syt1's membrane insertion is indeed critical for Ca^2+^-triggered opening of the fusion (Fig. 4), consistent with the conclusion of many previous *in vivo* and *in vitro* studies^7^,^25^. For Syt1 it is unclear if SNARE- and membrane-binding is sequential. Our previous work raised the possibility that Syt1 simultaneously binds the SNARE complex and the PIP_2_-containing membrane^30^,^39^. Here, based on experiments involving Syt1 mutants, we show this concurrent SNARE/PIP_2_ interaction through the polybasic region on Syt1 in the absence of Ca^2+^, might be essential for synaptic vesicle docking.

How might the Syt1's insertion into the membrane promote the opening of fusion-pores for content release? McMahon and Chapman have independently shown that Syt1 has the capacity to induce a positive curvature of the bilayer^33^,^50^. They demonstrated that this newly discovered function is well correlated with the ability to stimulate lipid mixing (or hemifusion) with Ca^2+^. It is also possible that this curvature-inducing ability of Syt1 might provide some thrust to overcome the energy barrier for pore opening. Alternatively, it has long been thought that transmembrane domains of fusion proteins, including those in viral membrane fusion as well as in intracellular membrane fusion, play a critical role in driving the fusion-pore^59^,^60^. It has been shown that Syt1 binds the basic membrane proximal linker region of SNAREs where PIP_2_ molecules can cluster^43^. We speculate that this interaction might activate transmembrane domains to drive fusion pore opening, although more work is needed to support this idea. Very recently, Südhof and coworkers have disputed the critical involvement of SNARE transmembrane domains for Ca^2+^-triggered exocytosis, warranting further work in this area^61^.

In summary, using a series of single vesicle assays^40^,^46^,^62^,^63^,^64^ resolving the individual fusion steps in the single vesicle fusion event, we have shown that Syt1-SNARE/PIP_2_ coupling through the polybasic region has little to do with content release although it plays a significant role in vesicle docking. On the other hand, our results demonstrate that Ca^2+^-induced insertion of Syt1 into the membrane is essential for content release. Our results thus delineate multiple functions of Syt1 along the pathway of Ca^2+^-triggered exocytosis.

## Methods

### Plasmid constructs and site-directed mutagenesis

DNA sequences encoding rat syntaxin 1A (amino acids 1–288 with three native cysteines C145, C271, and C272 replaced by alanines), rat VAMP2 (amino acids 1–116 with C103 replaced by alanine), rat SNAP-25 (amino acids 1–206 with four native cysteines C85, C88, C90, and C92 replaced by alanines), soluble syntaxin 1A (amino acid 191–266), and soluble VAMP2 (amino acids 1–96) were inserted into the pGEX-KG vector as N-terminal glutathione S-transferase (GST) fusion proteins. SNAP-25 was inserted into a pET-28b vector also as an N-terminal 6xHistidine (His)-tag fusion protein. Recombinant synaptotagmin 1 (Syt1, amino acids 50–421 with four native cysteines C74, C75, C77 and C79 replaced by alanines and another C82 replaced by serine, (we denoted this recombinant form as wild-type Syt1 in this work and used it as the template for generating the point mutants) was inserted into a pET-28b vector as a C-terminal His-tagged protein. It has been previously shown that the deleted first 49 N-terminal residues does not affect the Syt1 function in exocytosis^33^,^37^,^38^. The Quick Change site-directed mutagenesis kit (Stratagene) was used to generate all Syt1 mutants, including Syt1 EEE (L326/327/331E), Syt1 QQQ (L326/327/331Q), Syt1 Y311N, Syt1 2A(A) (M173A/F234A), Syt1 2A(B) (V304A/I367A), Syt1 4A (M173A/F234A/V304A/I367A), Syt1 2W(A) (M173W/F234W), Syt1 2W(B) (V304W/I367W), Syt1 4W (M173W/F234W/V304W/I367W), Syt1-C2A* (D230A/D232A), Syt1-C2B* (D363A/D365A), and Syt1-C2A*B* (D230A/D232A/D363A/D365A). DNA sequences were confirmed by the Iowa State University DNA Sequencing Facility.

### Protein expression and purification

The GST-tagged proteins were expressed in *E. coli* Rosetta (DE3) pLysS (Novagene). Details can be found in our previous work^39^,^65^. The His-tagged proteins were expressed in *E. coli* BL21 (DE3) (Novagen) and purified using previously-described protocol^39^,^66^.

### Membrane reconstitution

The lipid molecules used in this study were 1,2-dioleoyl-sn-glycero-3-phospho-L-serine (DOPS), 1-palmitoyl-2-oleoyl-sn-glycero-3-phosphocholine (POPC), phosphatidylinositol-4,5-bisphosphate (PIP_2_, from porcine brain), cholesterol, and 1,2-dipalmitoyl-sn-glycero-3-phosphoethanolamin-N-(biotinyl) (biotin-DPPE). All lipids were obtained from Avanti Polar Lipids. 1,1′-Dioctadecyl-3,3,3′,3′-Tetramethylindocarbocyanine Perchlorate (DiI), 1,1′-Dioctadecyl-3,3,3′,3′-Tetramethylindodicarbocyanine Perchlorate (DiD), and sulforhodamine B were obtained from Invitrogen.

For the single vesicle docking assay, the molar ratios of lipids were 15:61:20:2:2 (DOPS:POPC:cholesterol:PIP_2_:DiD) for the t-SNARE-reconstituted (t-)vesicles, and 5:73:20:2 (DOPS:POPC:cholesterol:DiI) for the v-SNARE-reconstituted (v-)vesicles, respectively. To fix v-vesicles on the TIR imaging surface coated with neutravidin, 0.1 mol% biotin-DPPE was added to the v-vesicle lipid mixture prior to the rehydration-freeze thaw-extrusion steps (see below). The lipid mixture was first completely dried and then rehydrated with dialysis buffer (25 mM HEPES, pH 7.4, 100 mM KCl). After five freeze–thaw cycles, protein-free large unilamellar vesicles (~100 nm in diameter) were obtained by extrusion through a 100 nm polycarbonate filter (Whatman). For membrane reconstitution, SNARE proteins and Syt1 were mixed with protein-free vesicles at a protein-to-lipid molar ratio of 1:200 for each protein component (this ratio was kept the same for all experiments, including the single vesicle content mixing assay) with ~0.8% OG in the dialysis buffer at 4°C for 15 min. The mixture was diluted by a factor of two with the dialysis buffer and this diluted mixture was then dialyzed overnight in 2 L dialysis buffer at 4°C. Details for the reconstitution process were discussed in our previous work^39^,^64^.

For the single vesicle content mixing assay using a small sulforhodamine B content indicator, lipid mixtures were prepared as described for the single vesicle docking assay except that the fluorescent lipid dyes (DiI and DiD) were replaced by equal amounts of POPC. The t-vesicles were prepared as described earlier, except that 50 mM sulforhodamine B in the dialysis buffer was kept throughout all the sample preparation steps for the v-vesicles. Free sulforhodamine B was removed through dialysis and a further PD-10 column desalting step (GE healthcare).

Membrane reconstitution efficiency was confirmed using liposome co-sedimentation assay followed by a SDS-page analysis. Briefly, the aggregated protein was first removed after dialysis by centrifugation at 10,000 g for 10 min at 4°C. Then the membrane-bound protein was pelleted through high-speed liposome sedimentation using an Airfuge Air-Driven Ultracentrifuge (Beckman) at 150,000 g for 30 min at 4°C. Pelleted vesicles were re-suspended in the dialysis buffer and re-subjected to the centrifuge for twice more. Finally, the pellets were re-suspended in 60 μl (about 1/3 of the initial volume) of dialysis buffer and analyzed by SDS-page.

### Single vesicle binding assay

Slide preparation was the same as that in the vesicle docking assay. SNARE-free vesicles (with or without 2 mol% PIP_2_) were immobilized on the PEG-coated surface. After two rounds of 200 μl dialysis buffer washing, Syt1 or its mutants reconstituted vesicles (Syt1-vesicles, DiD-labled without PIP2, 100 ~ 200 nM) were injected into the flow chamber for 30 min docking at room temperature. After washing out free Syt1-vesicles, the docked Syt1-vesicles number in the imaging area (45 × 90 μm^2^) was counted (Supplementary Figs. S10A-C).

### Single vesicle docking and content mixing assays

After coating the quartz surface with a solution of methoxy-polyethylene glycol (mPEG) and biotin-PEG molecules to eliminate non-specific binding of vesicles, the quartz slide was assembled into a flow chamber and coated with neutravidin (0.2 mg ml^−1^). Following 30 minutes of incubation at room temperature (~25 °C), the v-vesicles were immobilized on the PEG-coated surface. After two rounds of 200 μl dialysis buffer washing, t-vesicles (100 ~ 200 nM in lipid concentrations) were injected at room temperature into the flow chamber for 30 min of docking. After washing out free t-vesicles, we acquired images (45 × 90 μm^2^) from 5 ~ 40 random locations within the flow chamber using 635 nm laser excitation for docked t-vesicles and 532 nm laser excitation for immobilized v-vesicles, respectively. All spots appeared in the green channel were considered as docked t-vesicles. The spots in the image were identified by the smCamera program (kindly provided by Dr. Taekjip Ha's group) based on the criteria: peak radius, 3 pixel; peak threshold/data scaler, 1%. The docking probability was calculated as the ratio of average docked t-vesicles to average anchored v-vesicles (Supplementary Figs. S10D-F). Details of the single vesicle docking assay were reported in our previous work^67^,^68^.

For real-time imaging of small sulforhodamine B content release, sulforhodamine B containing v-vesicles was immobilized on the PEG-coated surface. After two rounds of washing using 1 ml dialysis buffer, t-vesicles were injected into the channel to make them bind to v-vesicles. After 30 min of incubation at room temperature (~25 °C), dialysis buffer, with or without 500 μM Ca^2+^, was injected into the flow chamber by a motorized syringe pump at a speed of 33 μl sec^−1^. The detail of the single vesicle content mixing assay was reported in previous work^45^,^46^,^62^.

### GST pull-down assay

To form a binary complex, His-tagged SNAP-25 and GST-tagged soluble Syntaxin 1A (191–266) cell lysates were mixed and loaded onto Ni-NTA beads. The binary complex was purified following the procedure described for His-tagged SNAP-25, and the purified binary complex was then loaded onto Glutathione-agarose beads. After washing thoroughly with a cleavage buffer (50 mM Tris-HCl, pH 8.0, 150 mM NaCl, 0.8% OG) to remove unbound His-tagged SNAP-25, the binary complex immobilized beads were separated into many equal parts in 1.7 ml Eppendorf tubes. Equal amounts of wild-type Syt1 or its mutants and cleavage buffer were added to the immobilized binary complex and the mixture was incubated at 4°C for 1 h. The beads were then thoroughly washed with the cleavage buffer. 5xSDS-loading buffer (313 mM Tris-HCl, pH 6.8, 10% SDS, 0.05% bromophenol blue, 50% glycerol, 0.5 M DTT) was added to the samples and the mixture was boiled for 10 min. Proteins were resolved on precast 12% SDS-page and visualized using Coomassie blue staining. To test the binding of wild-type Syt1 and its mutants to the ternary SNARE complex, purified soluble Syntaxin 1A (191–266) was mixed with His-tagged SNAP-25 and GST-tagged soluble VAMP2 (1–96) cell lysates before loading onto Ni-NTA beads. The ternary complex was purified following the same procedure described for His-tagged SNAP-25. The purified ternary complex was then loaded onto Glutathione-agarose beads. After washing thoroughly with cleavage buffer to remove unbound His-tagged SNAP-25 and the binary complex, binding of wild-type Syt1 or its mutants was performed following the procedure described above for the binary complex, except that 500 μM Ca^2+^ was added to the cleavage buffer.

### CD spectroscopy

The CD spectra were measured with an AVIV stop-flow Circular Dichroism spectropolarimeter at 190 to 260 nm using a cell with the 1 mm path-length. The sample containing 10 μM of either wild-type Syt1 or the Y311N mutant was measured at 25°C. For the correction of the baseline error, the signal from a blank run with PBS buffer (137 mM NaCl, 2.7 mM KCl, 10 mM Na_2_HPO_4_, and 2 mM KH_2_PO_4_) containing 0.8% OG was subtracted from all the experimental spectra.

## Author Contributions

Y.Lai, X.L. and Y.-K.S. designed the experiments, Y.Lai, X.L. and J.D. carried out the experiments, and Y.Lai, X.L. and Y.-K.S. wrote the paper.

## Supplementary Material

Supplementary Information Supplementary Information

## References

[b1] Südhof T. C. The synaptic vesicle cycle. Annu Rev Neurosci 27, 509–547 (2004). 15217342 10.1146/annurev.neuro.26.041002.131412

[b2] Poirier M. A. *et al.* The synaptic SNARE complex is a parallel four-stranded helical bundle. Nat Struct Biol 5, 765–769 (1998). 9731768 10.1038/1799

[b3] Sutton R. B., Fasshauer D., Jahn R. & Brunger A. T. Crystal structure of a SNARE complex involved in synaptic exocytosis at 2.4 A resolution. Nature 395, 347–353 (1998). 9759724 10.1038/26412

[b4] Weber T. *et al.* SNAREpins: minimal machinery for membrane fusion. Cell 92, 759–772 (1998). 9529252 10.1016/s0092-8674(00)81404-x

[b5] Stein A., Weber G., Wahl M. C. & Jahn R. Helical extension of the neuronal SNARE complex into the membrane. Nature 460, 525–528 (2009). 19571812 10.1038/nature08156 PMC3108252

[b6] Fernandez-Chacon R. *et al.* Synaptotagmin I functions as a calcium regulator of release probability. Nature 410, 41–49 (2001). 11242035 10.1038/35065004

[b7] Chapman E. R. Synaptotagmin: a Ca(2+) sensor that triggers exocytosis? Nat Rev Mol Cell Biol 3, 498–508 (2002). 12094216 10.1038/nrm855

[b8] Rizo J. & Rosenmund C. Synaptic vesicle fusion. Nat Struct Mol Biol 15, 665–674 (2008). 18618940 10.1038/nsmb.1450 PMC2519048

[b9] Jahn R. & Fasshauer D. Molecular machines governing exocytosis of synaptic vesicles. Nature 490, 201–207 (2012). 23060190 10.1038/nature11320 PMC4461657

[b10] Rizo J. & Südhof T. C. The membrane fusion enigma: SNAREs, Sec1/Munc18 proteins, and their accomplices--guilty as charged? Annu Rev Cell Dev Biol 28, 279–308 (2012). 23057743 10.1146/annurev-cellbio-101011-155818

[b11] Perin M. S., Brose N., Jahn R. & Südhof T. C. Domain structure of synaptotagmin (p65). J Biol Chem 266, 623–629 (1991). 1985919

[b12] Sutton R. B., Davletov B. A., Berghuis A. M., Südhof T. C. & Sprang S. R. Structure of the first C2 domain of synaptotagmin I: a novel Ca2+/phospholipid-binding fold. Cell 80, 929–938 (1995). 7697723 10.1016/0092-8674(95)90296-1

[b13] Shao X., Davletov B. A., Sutton R. B., Südhof T. C. & Rizo J. Bipartite Ca2+-binding motif in C2 domains of synaptotagmin and protein kinase C. Science 273, 248–251 (1996). 8662510 10.1126/science.273.5272.248

[b14] Ubach J., Zhang X., Shao X., Südhof T. C. & Rizo J. Ca2+ binding to synaptotagmin: how many Ca2+ ions bind to the tip of a C2-domain? EMBO J 17, 3921–3930 (1998). 9670009 10.1093/emboj/17.14.3921 PMC1170727

[b15] Gerona R. R., Larsen E. C., Kowalchyk J. A. & Martin T. F. The C terminus of SNAP25 is essential for Ca(2+)-dependent binding of synaptotagmin to SNARE complexes. J Biol Chem 275, 6328–6336 (2000). 10692432 10.1074/jbc.275.9.6328

[b16] Earles C. A., Bai J., Wang P. & Chapman E. R. The tandem C2 domains of synaptotagmin contain redundant Ca2+ binding sites that cooperate to engage t-SNAREs and trigger exocytosis. J Cell Biol 154, 1117–1123 (2001). 11551981 10.1083/jcb.200105020 PMC2150817

[b17] Zhang X., Kim-Miller M. J., Fukuda M., Kowalchyk J. A. & Martin T. F. Ca2+-dependent synaptotagmin binding to SNAP-25 is essential for Ca2+-triggered exocytosis. Neuron 34, 599–611 (2002). 12062043 10.1016/s0896-6273(02)00671-2

[b18] de Wit H. *et al.* Synaptotagmin-1 docks secretory vesicles to syntaxin-1/SNAP-25 acceptor complexes. Cell 138, 935–946 (2009). 19716167 10.1016/j.cell.2009.07.027

[b19] Vrljic M. *et al.* Molecular mechanism of the synaptotagmin-SNARE interaction in Ca2+-triggered vesicle fusion. Nat Struct Mol Biol 17, 325–331 (2010). 20173762 10.1038/nsmb.1764 PMC2928146

[b20] Bai J., Tucker W. C. & Chapman E. R. PIP2 increases the speed of response of synaptotagmin and steers its membrane-penetration activity toward the plasma membrane. Nat Struct Mol Biol 11, 36–44 (2004). 14718921 10.1038/nsmb709

[b21] Li L. *et al.* Phosphatidylinositol phosphates as co-activators of Ca2+ binding to C2 domains of synaptotagmin 1. J Biol Chem 281, 15845–15852 (2006). 16595652 10.1074/jbc.M600888200

[b22] Kuo W., Herrick D. Z., Ellena J. F. & Cafiso D. S. The calcium-dependent and calcium-independent membrane binding of synaptotagmin 1: two modes of C2B binding. J Mol Biol 387, 284–294 (2009). 19302798 10.1016/j.jmb.2009.01.064 PMC2669496

[b23] Radhakrishnan A., Stein A., Jahn R. & Fasshauer D. The Ca2+ affinity of synaptotagmin 1 is markedly increased by a specific interaction of its C2B domain with phosphatidylinositol 4,5-bisphosphate. J Biol Chem 284, 25749–25760 (2009). 19632983 10.1074/jbc.M109.042499 PMC2757977

[b24] van den Bogaart G., Meyenberg K., Diederichsen U. & Jahn R. Phosphatidylinositol 4,5-bisphosphate increases Ca2+ affinity of synaptotagmin-1 by 40-fold. J Biol Chem 287, 16447–16453 (2012). 22447935 10.1074/jbc.M112.343418 PMC3351315

[b25] Chapman E. R. How does synaptotagmin trigger neurotransmitter release? Annu Rev Biochem 77, 615–641 (2008). 18275379 10.1146/annurev.biochem.77.062005.101135

[b26] Rhee J. S. *et al.* Augmenting neurotransmitter release by enhancing the apparent Ca2+ affinity of synaptotagmin 1. Proc Natl Acad Sci U S A 102, 18664–18669 (2005). 16352718 10.1073/pnas.0509153102 PMC1311909

[b27] Lynch K. L. *et al.* Synaptotagmin-1 utilizes membrane bending and SNARE binding to drive fusion pore expansion. Mol Biol Cell 19, 5093–5103 (2008). 18799625 10.1091/mbc.E08-03-0235 PMC2592635

[b28] Lai A. L., Huang H., Herrick D. Z., Epp N. & Cafiso D. S. Synaptotagmin 1 and SNAREs form a complex that is structurally heterogeneous. J Mol Biol 405, 696–706 (2011). 21087613 10.1016/j.jmb.2010.11.015 PMC3039131

[b29] Vennekate W. *et al.* Cis- and trans-membrane interactions of synaptotagmin-1. Proc Natl Acad Sci U S A 109, 11037–11042 (2012). 22711810 10.1073/pnas.1116326109 PMC3390864

[b30] Kim J. Y. *et al.* Solution single-vesicle assay reveals PIP2-mediated sequential actions of synaptotagmin-1 on SNAREs. EMBO J 31, 2144–2155 (2012). 22407297 10.1038/emboj.2012.57 PMC3343461

[b31] Loewen C. A., Lee S. M., Shin Y. K. & Reist N. E. C2B polylysine motif of synaptotagmin facilitates a Ca2+-independent stage of synaptic vesicle priming in vivo. Mol Biol Cell 17, 5211–5226 (2006). 16987956 10.1091/mbc.E06-07-0622 PMC1679685

[b32] Gaffaney J. D., Dunning F. M., Wang Z., Hui E. & Chapman E. R. Synaptotagmin C2B domain regulates Ca2+-triggered fusion in vitro: critical residues revealed by scanning alanine mutagenesis. J Biol Chem 283, 31763–31775 (2008). 18784080 10.1074/jbc.M803355200 PMC2581593

[b33] Hui E., Johnson C. P., Yao J., Dunning F. M. & Chapman E. R. Synaptotagmin-mediated bending of the target membrane is a critical step in Ca(2+)-regulated fusion. Cell 138, 709–721 (2009). 19703397 10.1016/j.cell.2009.05.049 PMC2758036

[b34] Hui E. *et al.* Mechanism and function of synaptotagmin-mediated membrane apposition. Nat Struct Mol Biol 18, 813–821 (2011). 21642967 10.1038/nsmb.2075 PMC3130839

[b35] van den Bogaart G. *et al.* Synaptotagmin-1 may be a distance regulator acting upstream of SNARE nucleation. Nat Struct Mol Biol 18, 805–812 (2011). 21642968 10.1038/nsmb.2061 PMC3130798

[b36] Shin O. H., Rizo J. & Südhof T. C. Synaptotagmin function in dense core vesicle exocytosis studied in cracked PC12 cells. Nat Neurosci 5, 649–656 (2002). 12055633 10.1038/nn869

[b37] Yao J., Kwon S. E., Gaffaney J. D., Dunning F. M. & Chapman E. R. Uncoupling the roles of synaptotagmin I during endo- and exocytosis of synaptic vesicles. Nat Neurosci 15, 243–249 (2012). 22197832 10.1038/nn.3013 PMC3435110

[b38] Wang Z., Liu H., Gu Y. & Chapman E. R. Reconstituted synaptotagmin I mediates vesicle docking, priming, and fusion. J Cell Biol 195, 1159–1170 (2011). 22184197 10.1083/jcb.201104079 PMC3246889

[b39] Lai Y. & Shin Y. K. The importance of an asymmetric distribution of acidic lipids for synaptotagmin 1 function as a Ca2+ sensor. Biochem J 443, 223–229 (2012). 22229667 10.1042/BJ20112044 PMC4412275

[b40] Lai Y., Lou X., Jho Y., Yoon T. Y. & Shin Y. K. The synaptotagmin 1 linker may function as an electrostatic zipper that opens for docking but closes for fusion pore opening. Biochem J 456, 25–33 (2013). 24001110 10.1042/BJ20130949 PMC4418238

[b41] Xu Y., Zhang F., Su Z., McNew J. A. & Shin Y. K. Hemifusion in SNARE-mediated membrane fusion. Nat Struct Mol Biol 12, 417–422 (2005). 15821745 10.1038/nsmb921

[b42] Diao J. J., Zhao M. L., Zhang Y. X., Kyoung M. & Brunger A. T. Studying protein-reconstituted proteoliposome fusion with content indicators in vitro. Bioessays 35, 658–665 (2013). 23625805 10.1002/bies.201300010 PMC4453005

[b43] Honigmann A. *et al.* Phosphatidylinositol 4,5-bisphosphate clusters act as molecular beacons for vesicle recruitment. Nat Struct Mol Biol 20, 679–686 (2013). 23665582 10.1038/nsmb.2570 PMC3676452

[b44] Yoon T. Y. *et al.* Complexin and Ca2+ stimulate SNARE-mediated membrane fusion. Nat Struct Mol Biol 15, 707–713 (2008). 18552825 10.1038/nsmb.1446 PMC2493294

[b45] Diao J. *et al.* Synaptic proteins promote calcium-triggered fast transition from point contact to full fusion. eLife 1, e00109 (2012). 23240085 10.7554/eLife.00109 PMC3514886

[b46] Lai Y. *et al.* Fusion pore formation and expansion induced by Ca2+ and synaptotagmin 1. Proc. Natl. Acad. Sci. U S A 110, 1333–1338 (2013). 23300284 10.1073/pnas.1218818110 PMC3557091

[b47] Choi U. B. *et al.* Single-molecule FRET-derived model of the synaptotagmin 1-SNARE fusion complex. Nat Struct Mol Biol 17, 318–324 (2010). 20173763 10.1038/nsmb.1763 PMC2922927

[b48] Rickman C. *et al.* Conserved prefusion protein assembly in regulated exocytosis. Mol Biol Cell 17, 283–294 (2006). 16267273 10.1091/mbc.E05-07-0620 PMC1345666

[b49] Fernandez I. *et al.* Three-dimensional structure of the synaptotagmin 1 C2B-domain: synaptotagmin 1 as a phospholipid binding machine. Neuron 32, 1057–1069 (2001). 11754837 10.1016/s0896-6273(01)00548-7

[b50] Martens S., Kozlov M. M. & McMahon H. T. How synaptotagmin promotes membrane fusion. Science 316, 1205–1208 (2007). 17478680 10.1126/science.1142614

[b51] Lee H. K. *et al.* Dynamic Ca2+-dependent stimulation of vesicle fusion by membrane-anchored synaptotagmin 1. Science 328, 760–763 (2010). 20448186 10.1126/science.1187722 PMC2994549

[b52] Diao J. J. *et al.* Complexin-1 Enhances the On-Rate of Vesicle Docking via Simultaneous SNARE and Membrane Interactions. Journal of the American Chemical Society 135, 15274–15277 (2013). 24083833 10.1021/ja407392n PMC3854000

[b53] Tang J. *et al.* A complexin/synaptotagmin 1 switch controls fast synaptic vesicle exocytosis. Cell 126, 1175–1187 (2006). 16990140 10.1016/j.cell.2006.08.030

[b54] Schaub J. R., Lu X., Doneske B., Shin Y. K. & McNew J. A. Hemifusion arrest by complexin is relieved by Ca2+-synaptotagmin I. Nat Struct Mol Biol 13, 748–750 (2006). 16845390 10.1038/nsmb1124

[b55] Giraudo C. G. *et al.* Alternative zippering as an on-off switch for SNARE-mediated fusion. Science 323, 512–516 (2009). 19164750 10.1126/science.1166500 PMC3736854

[b56] Krishnakumar S. S. *et al.* A conformational switch in complexin is required for synaptotagmin to trigger synaptic fusion. Nat Struct Mol Biol 18, 934–940 (2011). 21785412 10.1038/nsmb.2103 PMC3668341

[b57] Yang X., Cao P. & Südhof T. C. Deconstructing complexin function in activating and clamping Ca2+-triggered exocytosis by comparing knockout and knockdown phenotypes. Proc Natl Acad Sci U S A 110, 20777–20782 (2013). 24297916 10.1073/pnas.1321367110 PMC3870694

[b58] Krishnakumar S. S. *et al.* Conformational dynamics of calcium-triggered activation of fusion by synaptotagmin. Biophys J 105, 2507–2516 (2013). 24314081 10.1016/j.bpj.2013.10.029 PMC3853086

[b59] Tong J., Borbat P. P., Freed J. H. & Shin Y. K. A scissors mechanism for stimulation of SNARE-mediated lipid mixing by cholesterol. Proc Natl Acad Sci U S A 106, 5141–5146 (2009). 19251653 10.1073/pnas.0813138106 PMC2663986

[b60] Hernandez J. M. *et al.* Membrane fusion intermediates via directional and full assembly of the SNARE complex. Science 336, 1581–1584 (2012). 22653732 10.1126/science.1221976 PMC3677693

[b61] Zhou P., Bacaj T., Yang X., Pang Z. P. & Südhof T. C. Lipid-anchored SNAREs lacking transmembrane regions fully support membrane fusion during neurotransmitter release. Neuron 80, 470–483 (2013). 24120845 10.1016/j.neuron.2013.09.010 PMC3872166

[b62] Kyoung M. *et al.* In vitro system capable of differentiating fast Ca2+-triggered content mixing from lipid exchange for mechanistic studies of neurotransmitter release. Proc Natl Acad Sci U S A 108, E304–313 (2011). 21705659 10.1073/pnas.1107900108 PMC3141984

[b63] Kyoung M., Zhang Y., Diao J., Chu S. & Brunger A. T. Studying calcium-triggered vesicle fusion in a single vesicle-vesicle content and lipid-mixing system. Nat Protoc 8, 1–16 (2013). 23222454 10.1038/nprot.2012.134 PMC3566647

[b64] Diao J. *et al.* A single vesicle-vesicle fusion assay for in vitro studies of SNAREs and accessory proteins. Nat Protoc 7, 921–934 (2012). 22582418 10.1038/nprot.2012.020 PMC4410872

[b65] Lai Y., Lou X., Wang C., Xia T. & Tong J. Synaptotagmin 1 and Ca2+ drive trans SNARE zippering. Sci Rep 4, 4575 (2014). 24694579 10.1038/srep04575 PMC3974132

[b66] Lai Y. *et al.* Nonaggregated alpha-Synuclein Influences SNARE-Dependent Vesicle Docking via Membrane Binding. Biochemistry 53, 3889–3896 (2014). 24884175 10.1021/bi5002536 PMC4075992

[b67] Yoon T. Y. *et al.* Complexin and Ca(2+) stimulate SNARE-mediated membrane fusion. Nature Structural & Molecular Biology 15, 707–713 (2008). 10.1038/nsmb.1446 PMC2493294 18552825

[b68] Diao J., Yoon T. Y., Su Z. L., Shin Y. K. & Ha T. C2AB: A Molecular Glue for Lipid Vesicles with a Negatively Charged Surface. Langmuir 25, 7177–7180 (2009). 19563216 10.1021/la901676e PMC2730783

[b69] Fuson K. L., Montes M., Robert J. J. & Sutton R. B. Structure of human synaptotagmin 1 C2AB in the absence of Ca2+ reveals a novel domain association. Biochemistry 46, 13041–13048 (2007). 17956130 10.1021/bi701651k PMC5975968

