# Supplementary material for: Molecular origins of synaptotagmin 1 activities on vesicle docking and fusion pore opening
Source: Sci Rep. 2015 Mar 20;5:9267. doi: 10.1038/srep09267 (PMC4366854; doi:10.1038/srep09267)
Supplement: Supplementary Information [file srep09267-s1.pdf]

Supplementary Information

Molecular origins of synaptotagmin 1 activities on vesicle docking and fusion pore opening

Ying Lai[1,#], Xiaochu Lou[1,#], Jiajie Diao[2], and Yeon-Kyun Shin[1,2,*]

[1]Department of Biochemistry, Biophysics & Molecular Biology, Iowa State University, Ames, Iowa 50011, USA.

[2]Center for Mitochondrial Biology and Medicine, The Key Laboratory of Biomedical Information Engineering of Ministry of Education, School of Life Science and Technology and Frontier Institute of Life Science, Frontier Institute of Science and Technology (FIST), Xi'an Jiaotong University, Xi'an 710049, P. R. China.

[3]Biomedical Research Institute, Korea Institute of Science and Technology (KIST), Hwarangno 14-gil 6, Seongbuk-gu, Seoul 136-791, South Korea.

[#]These authors contributed equally to this work.

[*]Corresponding author: Tel: +1 515 294-2530; Fax: +1 515 294-0453; Email: colishin@iastate.edu

Supplementary Figures

Supplementary Figure S1, Circular Dichroism for wild-type Syt1 and the Y311N mutant.

Supplementary Figure S2, GST pull-down assay in the present of IP_3 and BSA.

Supplementary Figure S3, Membrane reconstitution efficiencies.

Supplementary Figure S4, Single-vesicle membrane binding and docking assays.

Supplementary Figure S5, Membrane binding assays (binding to PIP_2).

Supplementary Figure S6, Single-vesicle docking in the present of IP_3 and BSA.

Supplementary Figure S7, Membrane binding assays in the present of Ca^{2+}.

Supplementary Figure S8, Single-vesicle content-mixing experiments.

Supplementary Figure S9, Single-vesicle content-mixing ability of Syt1 at different concentrations.

Supplementary Figure S10, Data analysis strategy in single-vesicle membrane binding and docking assays.

Supplementary Tables

Supplementary Table S1, Single vesicle-vesicle docking assay with t-SNARE-vesicles and v-SANRE/Syt1-vesicles.

Supplementary Table S2, Numbers of content mixing events using the small content indicator, sulforhodamine B in the absence and presence of Ca^{2+}.

Supplementary Figures

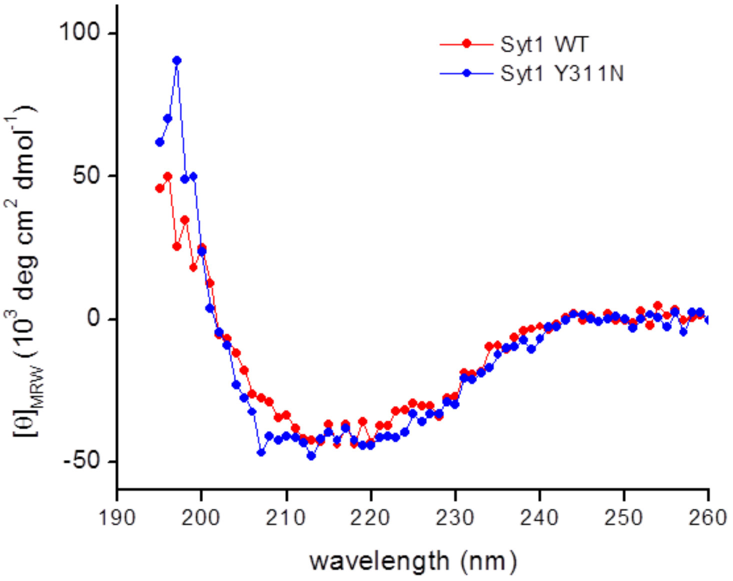

Supplementary Figure S1. The CD spectra for wild-type Syt1 (red) and Syt1 Y311N (blue) show no major changes of the secondary structure by the Tyr to Asn mutation at position 311.

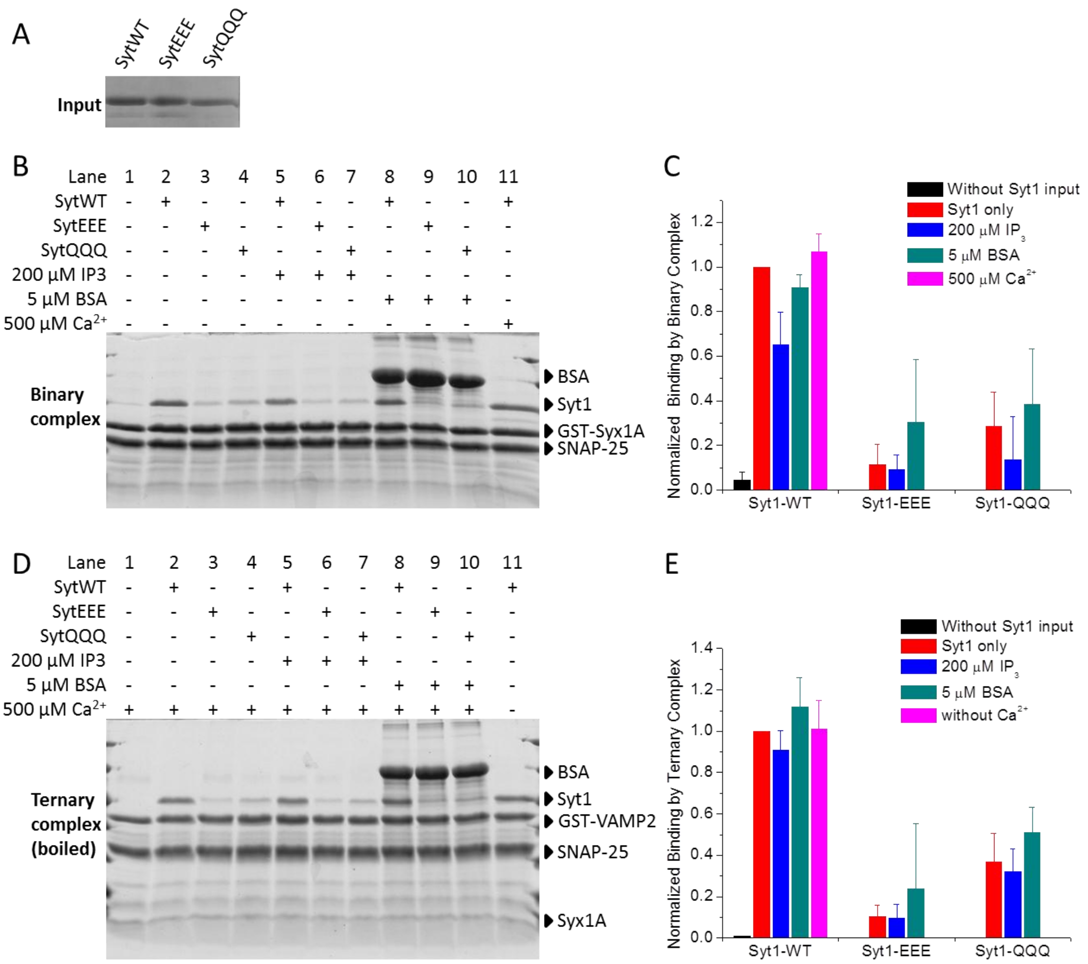

Supplementary Figure S2. GST pull-down assay in the present of IP_3 and BSA. (A) Analysis of input wild-type Syt1 and its polybasic region mutants (EEE and QQQ) on SDS-page. (B) SDS-page analysis of the GST pull-down assay between the binary t-SNARE complex and Syt1. (C) Normalized binding abilities of Syt1 and its mutants to the binary t-SNARE complex. (D) SDS-page analysis of the GST pull-down assay between the ternary SNARE complex and Syt1. (E) Normalized binding abilities of Syt1 and its mutants to the ternary t-SNARE complex. The experiments were repeated two times independently and samples from each trial are analyzed twice by SDS-page. Image acquisition and band analysis were performed using the ChemiDOC system (Bio-Rad). Results shown represent the mean ± S.D. We note that error bars for EEE and QQQ in the presence of BSA is large, which may be due to the smearing of BSA bands.

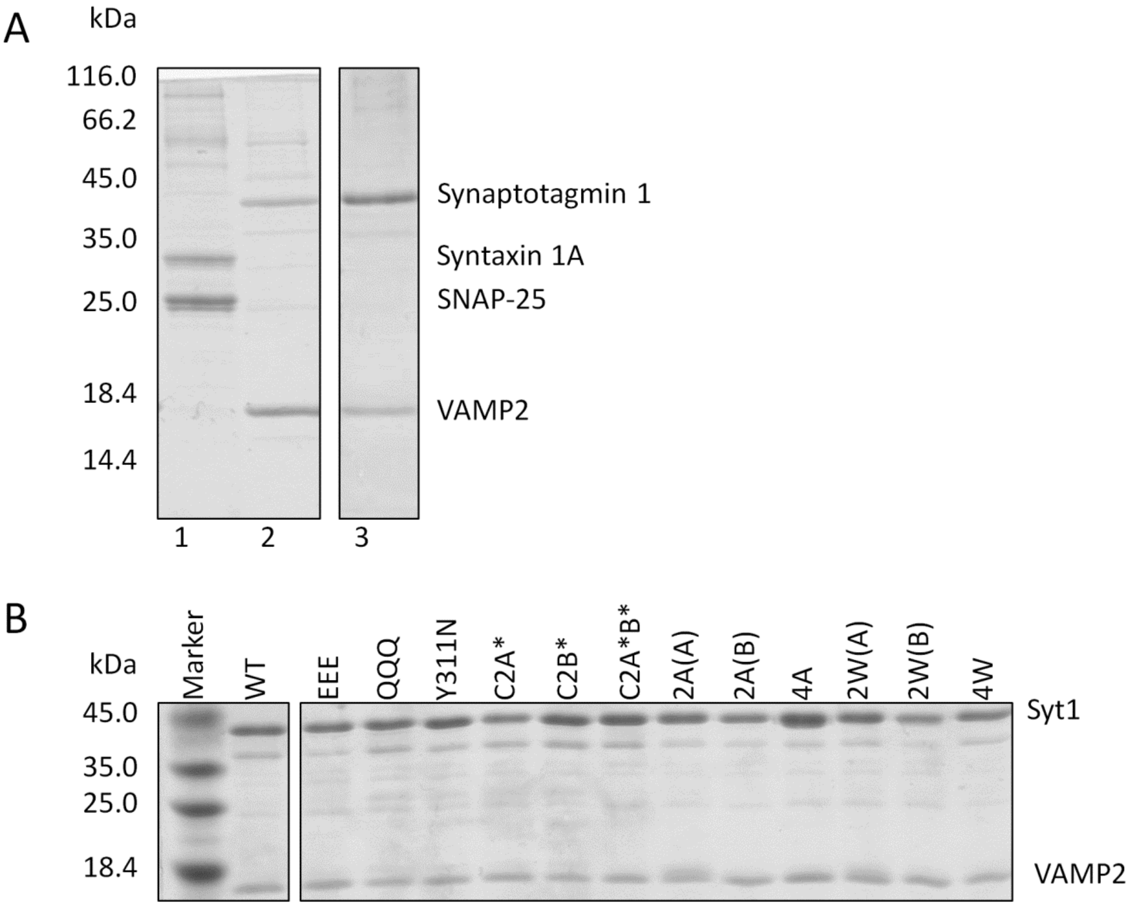

Supplementary Figure S3. Membrane reconstitution. (A) Analysis of reconstituted t-vesicles and v-vesicles on SDS-page. After reconstitution, the t-vesicles contain t-SNAREs (Syntaxin 1A: SNAP-25) at nearly 1:1 molar ratio (lane 1), and the v-vesicles contain Syt1 and VAMP2 at 1:4 molar ratio (lane 2) or 1:1 molar ratio (lane 3). SNARE proteins were kept at the protein-to-lipid ratio of 1:200 unless otherwise noted. (B) Membrane reconstitution efficiencies of wild-type Syt1 and its mutants together with VAMP2 at 1:1 molar ratio.

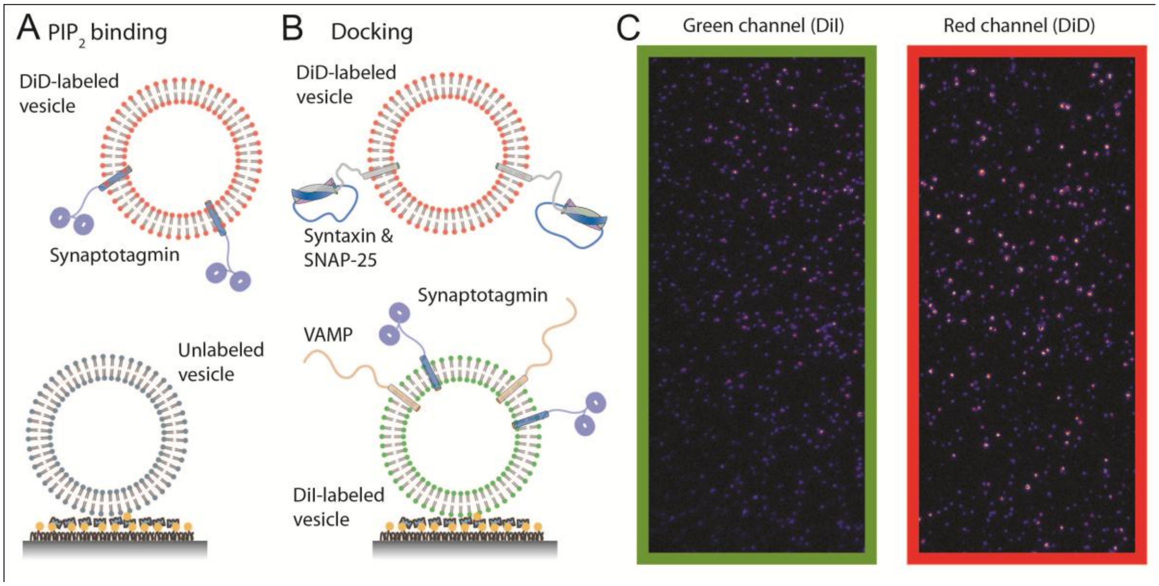

Supplementary Figure S4. Single-vesicle membrane binding and docking assays. (A) Schematic diagrams of the membrane binding (A) and single-vesicle docking (B) assays. (C) Imaging area (45×90 μm^2) after washing out free DiD-labeled vesicles. Each spot in the left channel represents a single DiI-labeled vesicle excited by green laser (532 nm), and each spot in the right channel represents a single DiD-labeled vesicle excited by red laser (635 nm).

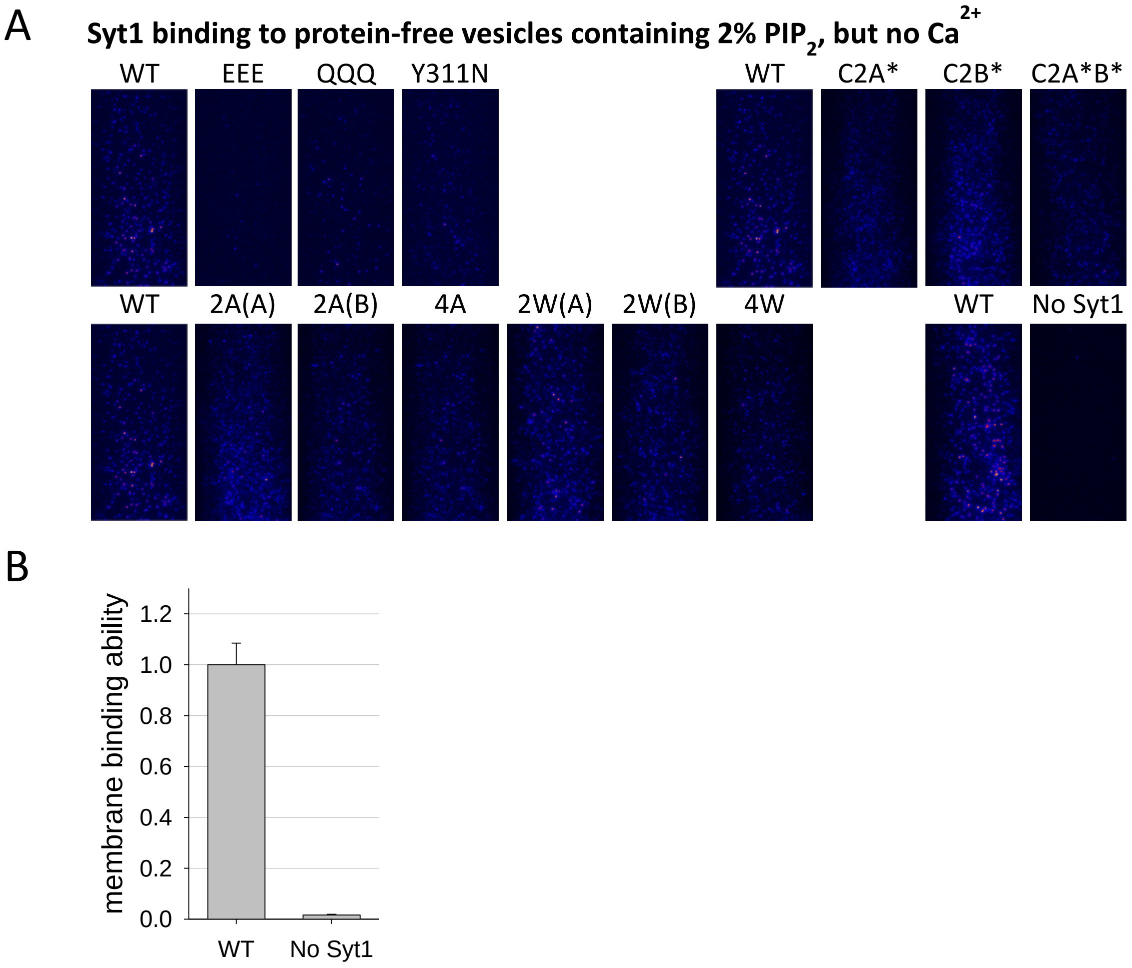

Supplementary Figure S5. Syt1 binding to the membrane through the polybasic region in the absence of Ca^{2+}. (A) The representative images of docked DiD-labeled vesicles reconstituted with Syt1 or its mutants to the PIP_2 containing SNARE-free vesicles in the abscence of Ca^{2+} (red channel, Fig. S2C). (B) Bar graph of membrane binding abilities of DiD-labeled vesicles with or without wild-type Syt1. Membrane binding ability is defined as normalized count number of membrane-bound vesicles. Results shown represent the mean ± S.D. (n=3).

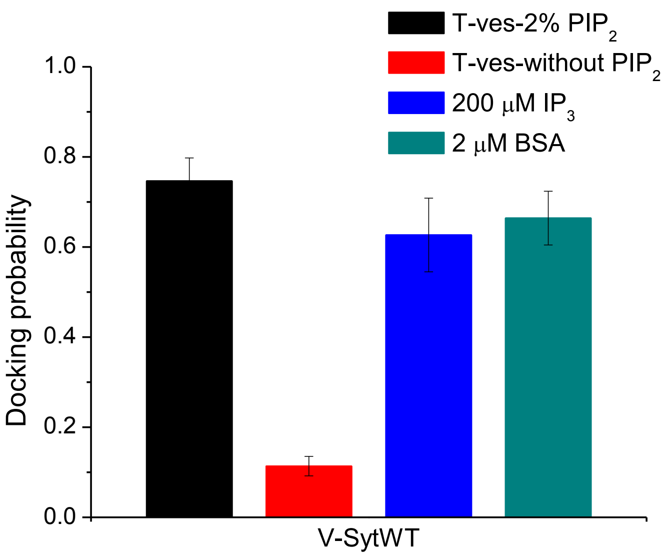

Supplementary Figure S6. Single-vesicle docking in the present of IP_3 and BSA. Single-vesicle docking probabilities of immobilized v-vesicles containing VAMP2 and wild-type Syt1 to t-vesicles containing 2% PIP_2 (black bar, as the same lipid composition as Figure 2A), t-vesicles without PIP_2 (red bar, replacing PIP_2 with the equal amount of POPC), t-vesicles containing 2% PIP_2 in the presence of 200 μM IP_3 (blue bar), and t-vesicles containing 2% PIP_2 in the presence of 2 μM BSA (dark cyan bar). Results shown represent the means ± S.D. from at least 10 screens in three independent measurements.

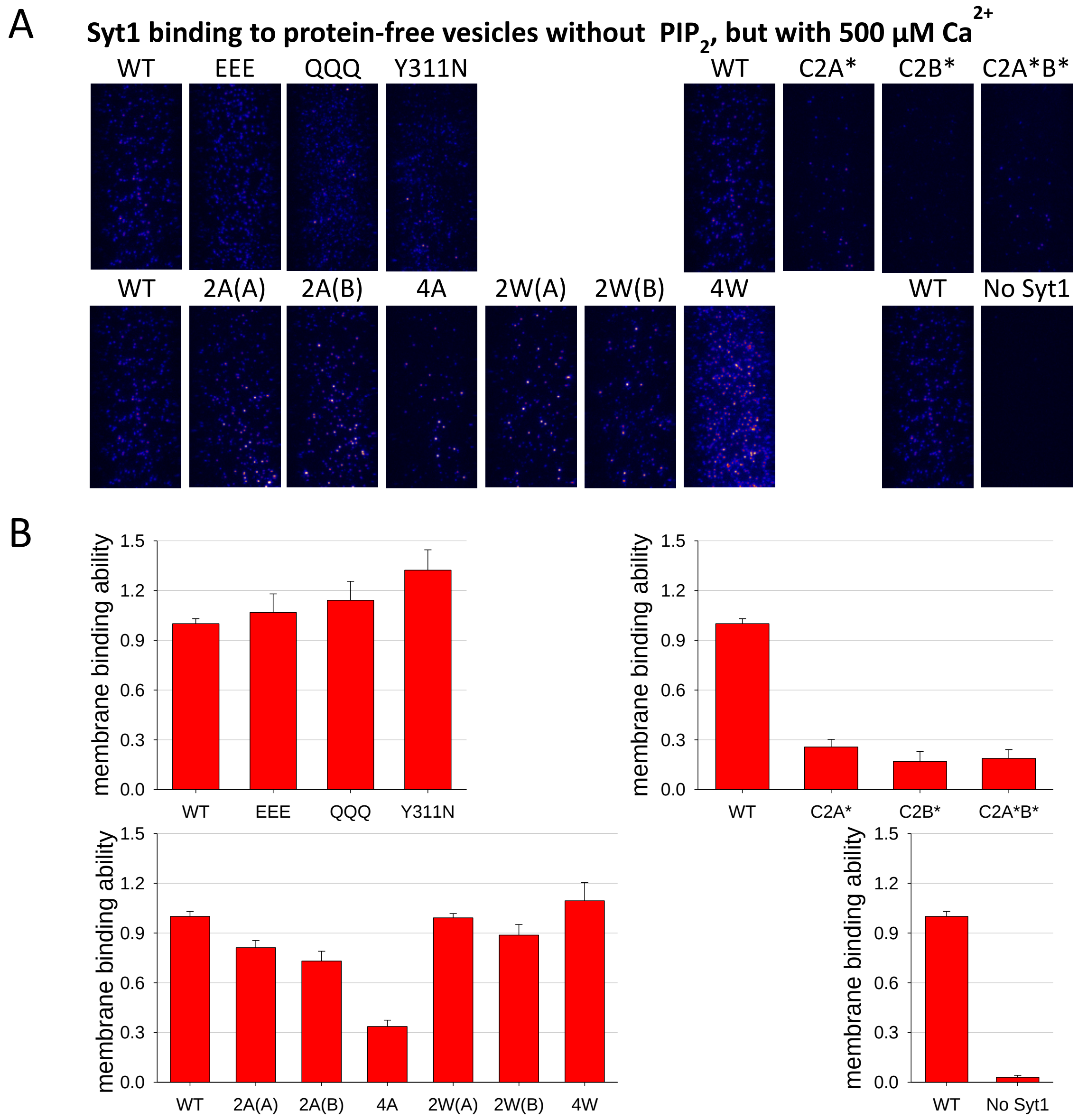

Supplementary Figure S7. Syt1 binding to the membrane through Ca^{2+} binding sites and the loop region in the presence of Ca^{2+}. (A) The representative images of docked DiD-labeled vesicles reconstituted with wild-type Syt1 or its mutants to SNARE-free vesicles (no PIP_2) in the prescence of 500 μM Ca^{2+} (red channel, Fig. S2C). (B) Bar graphs of membrane binding abilities in the presence of 500 μM Ca^{2+} for wild-type Syt1 and its mutants altered at the polybasic region, Ca^{2+} binding sites, and the loop region, and a bar graph of membrane binding

abilities of DiD-label vesicles with or without wild-type Syt1 (bottom right). Membrane binding ability is defined as normalized count number of membrane-bound vesicles. Results shown represent the mean ± S.D. (n=3).

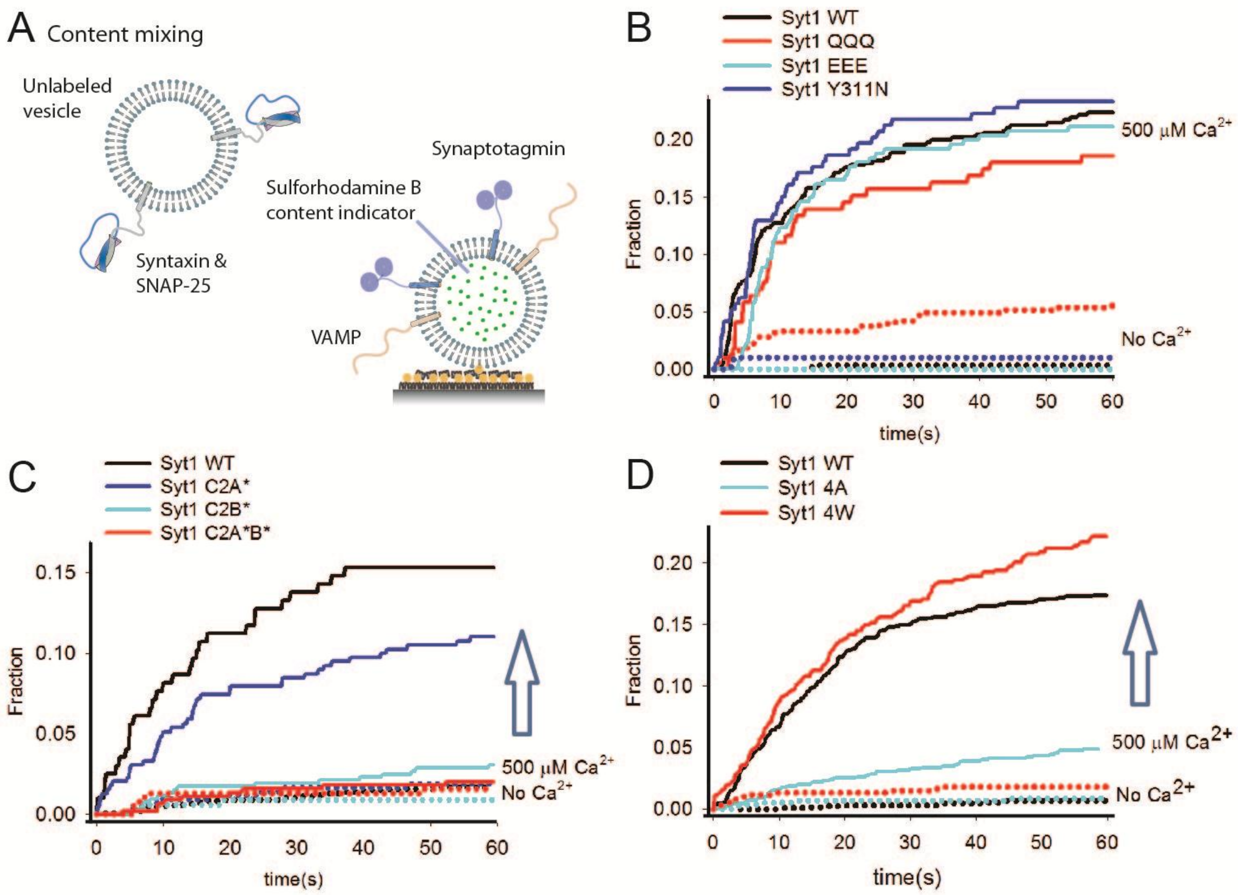

Supplementary Figure S8. Single-vesicle content-mixing experiments. (A) Schematic illustration of the single-vesicle content-mixing assay. Cumulative counts of vesicle fusion events in real time that show content mixing for wild-type Syt1 (black lines) and its polybasic region mutants (red lines for QQQ, cyan lines for EEE, and blue lines for Y311N) (B), its Ca^{2+} binding site mutants (red lines for C2A*B*, cyan lines for C2B*, and blue lines for C2A*) (C), and its loop region mutants (red lines for 4W and cyan lines for 4A) (D). The solid and dotted lines represent experiments with and without 500 μM Ca^{2+}, respectively. The cumulative time plots for wild-type Syt1 in (B)-(D) are slightly different from one another, indicating the variation among different batches of experiments.

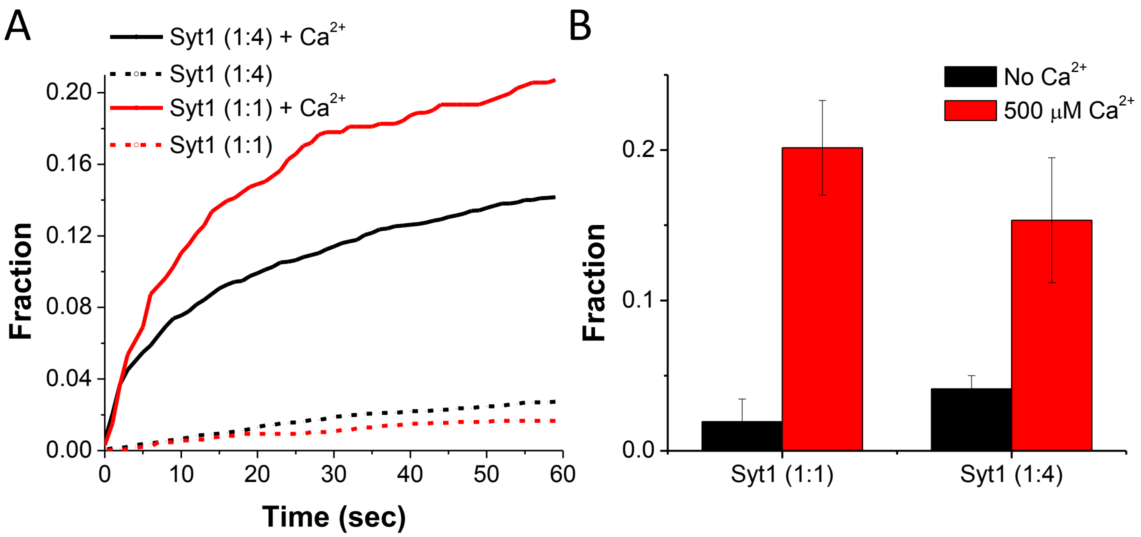

Supplementary Figure S9. Single-vesicle content-mixing activities at different Syt1 concentrations. (A) Cumulative counts of vesicle fusion events in real time that show content mixing between t- and v-vesicles incorporated with wild-type Syt1 and VAMP2 at 1:1 (solid red line) or 1:4 molar ratios (solid black line) in the presence of 500 μM Ca^{2+}. The dashed lines represent experiments in the absence of Ca^{2+}. (B) The bar graph represents fusion pore opening probabilities in first 60 seconds of the fusion reaction. Black bars are without Ca^{2+} while red bars are with 500 μM Ca^{2+}. Results shown represent the mean ± S.D. (n=3).

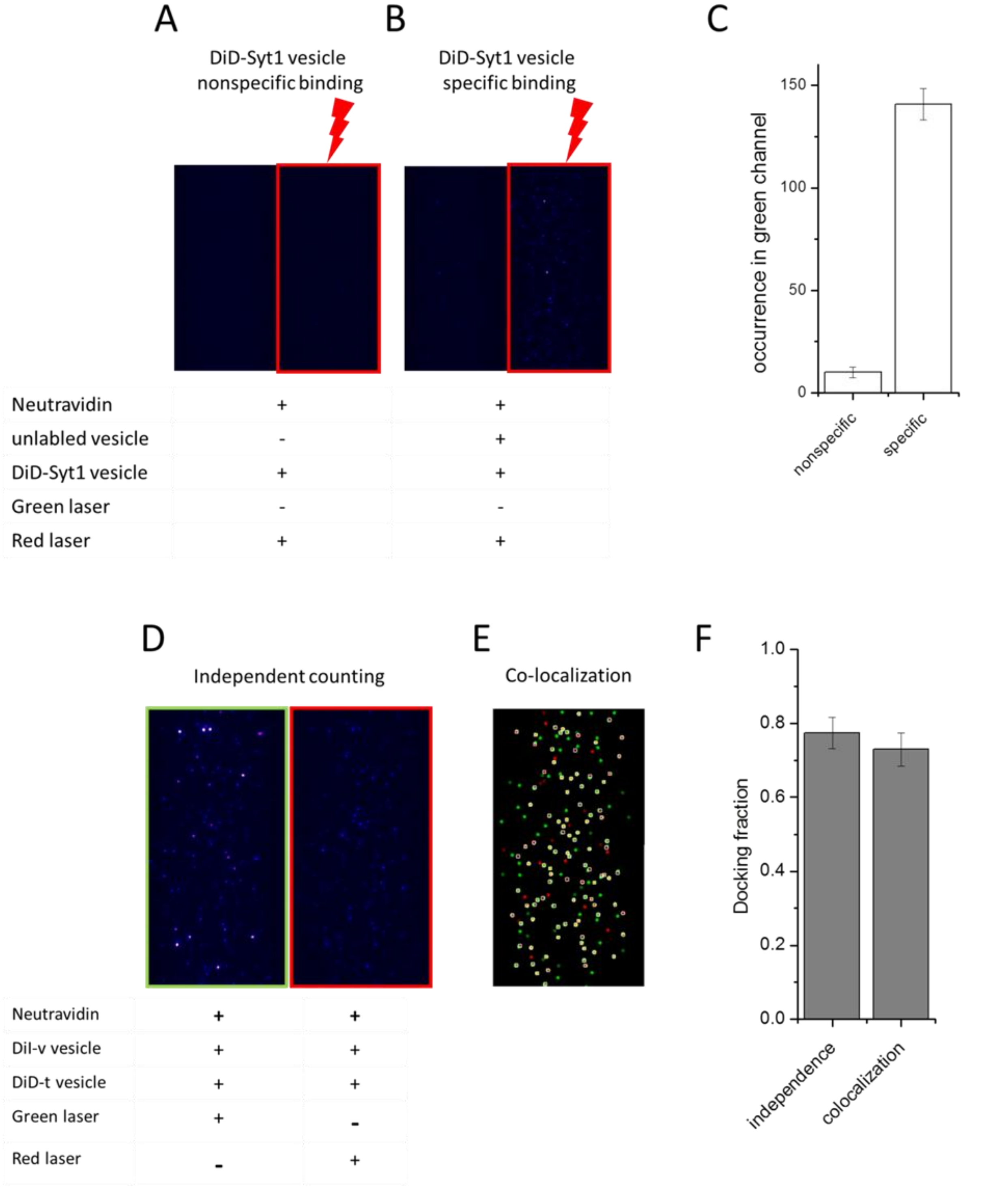

Supplementary Figure S10. Data analysis strategy in single-vesicle membrane binding and docking assays. (A-C) Non-specific and specific binding of DiD-Syt1 vesicle to PEG surface. (A) Non-specific binding of DiD-Syt1 vesicle to neutravidin surface is not significant. (B)

Specific binding of DiD-Syt1 vesicles to surface-immobilized SNARE-free unlabeled vesicles. (C) Bar graph of non-specific and specific binding. Results shown represent the mean ± S.D. (n=5). (D-F) No significant difference of docking probabilities between independent counting and co-localization counting. (D) Independent counting of immobilized DiI-v-vesicles by green laser excitation and that of docked DiD-t-vesicles by red laser excitation. (E) Co-localization counting. The green and red channels were overlapped through the smCamera program-generated map files. The white circles indicate co-localized vesicles. (F) Docking fraction obtained from independent and co-localization counting methods. Results shown represent the means ± S.D. (n=10).

Supplementary Table S1. Single vesicle-vesicle docking assay with t-SNARE-vesicles and v-SNARE/Syt1-vesicles. (# of screens means the number of images taken for analysis.)

	Docked t-vesicles/ # of screens	Docked t-vesicles per screen	Immobilized v-vesicles/ # of screens	Immobilized v-vesicles per screen	Docking percentage (%)
Syt1 WT	2119/10	212	2622/10	262	84.2
EEE	2686/40	67	4704/16	294	22.8
QQQ	3394/20	170	2632/8	329	51.7
Y311N	2733/60	46	2600/8	325	14.2
C2A*	1396/5	279	1886/5	377	74.0
C2B*	1462/10	146	2277/11	207	70.5
C2A*B*	2109/10	211	3630/11	330	63.9
2A(A)	2952/10	295	3512/8	439	67.2
2A(B)	2522/10	252	3304/8	413	61.0
4A	1678/10	168	2021/10	202	83.2
2W(A)	1588/10	159	2770/10	277	57.4
2W(B)	1897/10	190	3311/11	301	63.1
4W	1323/10	132	1812/10	181	72.9

Supplementary Table S2. Numbers of content mixing events using the small content indicator, sulforhodamine B in the absence and presence of Ca^{2+}

	No Ca^{2+} content mixing events	Number of t-docked vesicles	No Ca^{2+} content mixing percentage (%)	500 μM Ca^{2+} content mixing events	Number of t-docked vesicles	500 μM Ca^{2+} content mixing percentage (%)
Syt1 WT	17	1102	1.04	215	1177	17.18
EEE	42	1878	2.24	246	1315	18.71
QQQ	90	2007	4.48	345	1933	17.85
Y311N	9	1001	0.90	429	2302	18.64
C2A*	24	1254	1.91	129	1181	10.92
C2B*	9	999	0.90	48	1599	3.00
C2A*B*	18	1146	1.57	27	1352	2.00
2A(A)	6	907	0.66	324	3136	10.33
2A(B)	15	983	1.53	210	1435	14.63
4A	14	975	1.44	135	2815	4.80
2W(A)	12	847	1.42	234	1519	15.40
2W(B)	6	594	1.01	264	1378	19.16
4W	34	737	4.61	414	1882	22.00
